# Intention-to-Treat Analysis of Hepatic Resection for Liver Metastases from Uveal Melanoma: A Single-Center Experience

**DOI:** 10.1245/s10434-025-17115-0

**Published:** 2025-04-17

**Authors:** Marianna Maspero, Isabella Pezzoli, Lorenza Di Guardo, Martina Angi, Silvia Lo Dico, Carlo Sposito, Carlo Battiston, Vincenzo Mazzaferro

**Affiliations:** 1 HPB Surgery, Hepatology and Liver Transplantation Unit, Fondazione IRCCS Istituto Nazionale Tumori, Milan, Italy; 2 Department of Oncology and Hemato-Oncology, University of Milan, Milan, Italy; 3 Medical Oncology, Fondazione IRCCS Istituto Nazionale Tumori, Milan, Italy; 4 Ophthalmology, Fondazione IRCCS Istituto Nazionale Tumori, Milan, Italy; 5 Palliative Care, Fondazione IRCCS Istituto Nazionale Tumori, Milan, Italy

**Keywords:** Liver resection, Choroidal melanoma, Liver metastasis, Surgical oncology

## Abstract

**Background:**

Uveal melanoma metastasizes to the liver in almost 50% of patients. The prognosis of liver metastases from uveal melanoma (LMUM) is dismal; however, results from retrospective monocentric series suggest that surgery may improve survival in selected patients. We report our results of surgical explorations and hepatic resections for LMUM.

**Methods:**

We retrospectively analyzed our prospectively collected institutional database of hepatic resections for LMUM between 2013 and 2023.

**Results:**

In total, 22 patients underwent surgical exploration (median age 61 years, 41% female patients): 15 (68%) underwent curative-intent surgery (surgery group) while 7 (32%) had their resection aborted owing to intraoperatively unresectable disease (staging alone group). Patients in the surgery group had a longer interval between diagnosis of the primary tumor and LMUM (> 36 months in 64%, versus 29% in the staging alone group, *p* = 0.18). Preoperative imaging and intraoperative staging were concordant in ten (45%) cases, while five (23%) had more lesions, six (27%) had miliary disease, and one (4.5%) had peritoneal carcinomatosis. Minor postoperative complications occurred in 3 (14%) patients. All patients in the staging alone group underwent subsequent systemic or locoregional treatment. Median overall survival was 27 (15–47) months after surgery and 15 (8–21) months after staging alone. Recurrence after surgery occurred in 7/15 patients, with a median disease-free survival of 28 (4–38) months.

**Conclusions:**

More than 30% of patients with LMUM with preoperatively resectable disease are unresectable at explorative surgery. Acceptable recurrence rates and good survival outcomes are observed when radical surgery can be performed.

Uveal melanoma (UM) is the most common intraocular malignancy. Almost 50% of uveal melanomas metastasize, and the liver is the most common and exclusive site in up to 89% of patients.^1^ Most of these patients will die from metastatic evolution within 2 years.^2^

The management of liver metastases from uveal melanoma (LMUM) is complex, as this is a rare malignancy with limited reports in literature and limited treatment options.^3,4^ Differing from cutaneous melanoma, systemic treatment with immunotherapy is ineffective in UM, and, despite the development of new drugs such as the immune-mobilizing monoclonal T cell receptor tebentafusp,^5^ the prognosis with systemic treatment alone remains dismal.^4,6^

Reports on surgical treatment for LMUM are scarce. Evidence from these studies, however, suggests that the highest survival advantage for liver-limited LMUM is given by curative-intent hepatic resection (with or without ablation).^7,8^ As reported by Servois et al.,^9^ surgery for LMUM may be even offered as a second-line therapy after recurrence, and may even achieve cure in extremely selected cases; thus, aggressiveness appears warranted in patients with good performance status. Nonetheless, the tumor burden found intraoperatively is often higher than that reported by preoperative imaging, especially in the case of miliary disease.^10^ Consequently, patients may be exposed to the risk of unnecessary surgical explorations.

In this study, we report our 10-year monocentric experience of surgical exploration and hepatic resections for LMUM, focusing on concordance with preoperative imaging, postoperative complications, and long-term outcomes.

## Patients and Methods

### Study Population and Design

This was a retrospective, monocentric cohort study of surgical exploration and hepatic resection for LMUM. We retrospectively analyzed our prospectively collected database of surgical explorations for LMUM at our center between January 2013 and December 2023. All patients with disease that was preoperatively considered amenable to curative treatment were included, regardless of whether they ultimately underwent either curative-intent surgery (surgery group) or diagnostic laparoscopy/laparotomy with abandonment of curative-intent surgery owing to unresectable disease (staging-alone group). Inclusion criteria were: (1) age ≥ 18 years; (2) radiologic diagnosis of LMUM with at least one measurable lesion according to the Response Evaluation Criteria in Solid Tumors (RECIST) guidelines;^11^ (3) resectable disease at preoperative staging, undergoing laparoscopy or laparotomy with the intention of curative resection/ablation; and (4) any previous treatment to the primary tumor or to UM metastases.

The study was conducted according to the Declaration of Helsinki and its amendments. It was approved by our Institutional Review Board and reported according to Strengthening the Reporting of Observational Studies in Epidemiology (STROBE) guidelines.^12^

### Preoperative Staging, Patient Selection, and Operative Technique

All patients with UM treated at our center underwent a contrast-enhanced computed tomography (ceCT) scan of the chest, abdomen, and pelvis before treatment of the primary tumor to screen for distant metastases. After treatment of the primary tumor, they underwent follow-up with ceCT every 4 months, and, in case of suspicious liver lesions, contrast-enhanced magnetic resonance imaging (MRI) was performed. Fluorodeoxyglucose-positron emission tomography (FDG-PET) was not routinely used.

Patients with LMUM were discussed at the UM multidisciplinary tumor board and were proposed for surgical exploration if they had four or fewer resectable liver lesions on preoperative imaging. Usually, only patients with occurrence of metastatic disease at least 24 months after their initial diagnosis are considered for surgery. These recommendations are based on previous literature.^8^ Exceptions might have been made for young patients with liver lesions that were easily surgically approachable, especially if ablation could not be offered owing to tumor location. For patients with LMUM outside surgical criteria, we offered either locoregional therapies (transarterial chemoembolization and radioembolization), systemic treatment, or a combination of both.

After the indication for surgical exploration was given, patients underwent exploratory laparoscopy. If they were found to be resectable (i.e., no miliary disease, no peritoneal carcinomatosis, or no extrahepatic spread), we proceeded with curative-intent liver resection, either laparoscopically or with conversion to laparotomy, as necessary.

### Follow-up

After curative-intent resection, patients underwent follow-up in the UM multidisciplinary clinic with MRI and/or ceCT scan every 4 months for the first 2 years, then every 6 months thereafter. Recurrence was defined as the radiological and/or histological appearance of metastases from UM. In the case of recurrence, patients underwent multidisciplinary discussion and were treated as per standard of care.

### Collected Variables

Collected variables included demographics (age, sex, body mass index (BMI), comorbidities), characteristics of the primary tumor (date of diagnosis, TNM stage, treatment), characteristics of the LMUM (size, number, date of diagnosis), perioperative variables (date of surgery, type of surgery, operative time, postoperative length of stay, 90-day postoperative complications), treatment of LMUM for the staging-alone group, long-term outcomes (overall survival (OS), disease-free survival (DFS), site, and treatment of UM recurrence). Postoperative complications were graded according to the Clavien–Dindo classification.^13^

### Statistical Analysis

Descriptive variables were reported, with categorical variables expressed as numbers (percentages) and numerical variables as medians (interquartile ranges). The surgery group and staging-alone group were compared using Fisher’s exact test and Student’s *t*-test, as appropriate. The median follow-up was calculated using the inverse Kaplan–Meier method. The OS was calculated using the Kaplan–Meier method, starting at date of surgery and with censoring at death or last follow-up. For the surgery group, DFS was calculated using the Kaplan–Meier method, starting at the date of surgery with censoring at any UM recurrence or last follow-up. Postrecurrence survival (PRS) was calculated from the date of recurrence to the date of death or last follow-up. All analyses were conducted using IBM SPSS Statistics for Windows, version 26 (IBM Corp., Armonk, N.Y., USA).

## Results

### Demographics and Preoperative Treatment

Between 2013 and 2023, 22 patients with LMUM were taken to the operating room for curative-intent liver resection at our center. Their pre- and perioperative characteristics are shown in Table 1. In total, 15 (68%) underwent curative-intent resection as planned (surgery group), while 7 (32%) were found to have unresectable disease during surgical exploration (staging alone group). Treatment of the primary tumor was enucleation in 6 (27%) cases and radiotherapy in 16 (73%) cases; enucleation was more common in the surgery group (33% versus 14%, *p* = 0.262). Although not significant, the interval between diagnosis of the primary tumor and LMUM development was longer in the surgery group, with 64% being > 36 months (versus 29% in the staging alone group, *p* = 0.182).

**Table 1:** Preoperative and perioperative variables

	All (*n* = 22)	Surgery (*n* = 15)	Staging alone (*n* = 7)	*P* value
Age at surgery (years)	61 (47–69)	58.5 (38–69)	61 (55–70)	0.163
Sex	F:M 9 (41%):13 (59%)	F:M 5 (33%): 10 (67%)	F:M 4 (57%): 3 (43%)	0.376
ASA score				
I–II	16 (73%)	12 (79%)	4 (57%)	0.262
III–IV	6 (27%)	3 (21%)	3 (43%)	
Primary tumor treatment				
Enucleation	6 (27%)	5 (33%)	1 (14%)	0.674
Radiotherapy	16 (73%)	10 (67%)	6 (86%)	
Brachytherapy	1/16	1/10	0	
Proton beam RT	11/16	3/10	5/6	
Cyber/gamma knife	4/16	6/10	1/6	
Time between diagnosis of primary and liver metastasis, months	38 (26–66.5)	44.5 (29–79)	28 (25–44)	0.314
Time > 36 mo	11 (52%)	9 (64%)	2 (29%)	0.182
Previous hepatic locoregional treatments	4 (19%) 3 RFA, 1 TACE	4 (29%)	0	0.255
Preoperative staging				
Max size (mm)	24 (14–35)	16 (12.5–25.5)	20 (15.5–36)	0.926
Number	1 (1–2.5)	1 (1–2)	2 (1–3)	0.609
Bi-lobar	7 (33%)	3 (21%)	4 (57%)	0.156
Intraoperative staging				
Concordance with preoperative imaging	10 (45%)	10 (66%)	6 (86%)	0.165
More lesions	5 (23%)	5 (33%)	1 (14%)	
Miliary disease	6 (27%)			
Peritoneal carcinosis	1 (4.5%)			
Laparoscopic surgery	13 (59%)	7 (47%)	6 (86%)	
Histology				
Max size (mm)		15 (12–38)	–	
Number		2 (1–5)		
Morbidity	3 (14%)	3 (20%)	0	0.523
CD max	All II			
Length of stay, days	4.5 (4–7)	6 (4–7)	3.5 (3–4)	0.009

All data are numbers (percentages) and medians (interquartile ranges), as appropriate

*TACE* transarterial chemoembolization, *RFA* radiofrequency ablation, *CD* Clavien–Dindo classification, *mo* months

Four patients had already received liver-directed therapies for LMUM before surgery; three had received a percutaneous radiofrequency ablation, while one had received a transarterial chemoembolization (TACE). Liver-directed therapies were not offered within a downstaging protocol. Percutaneous radiofrequency ablation is preferred over surgery if surgery requires an extensive parenchymal loss; however, TACE was offered in a patient who did not fit criteria for surgery at the time. Three of those four liver-directed therapies occurred less than 6 months before surgical exploration, while the fourth patient received radiofrequency ablations 7, 4, and 1 year before surgery. All four successfully underwent curative-intent surgery.

### Surgical Exploration and Early Postoperative Period

All patients underwent laparoscopic exploration, except one patient who had to be explored laparoscopically owing to adherences from previous surgery. Following exploration and an intraoperative liver ultrasound, in 10 (45%) cases, the intraoperative picture was concordant with preoperative imaging. In addition, 5 (23%) patients had a higher number of lesions than in preoperative imaging but could still be resected; 6 (27%) had miliary disease; and one (5%) had peritoneal carcinomatosis. In the latter cases, liver resection was aborted and only staging and biopsy of the lesions were performed.

**Fig. 1:**
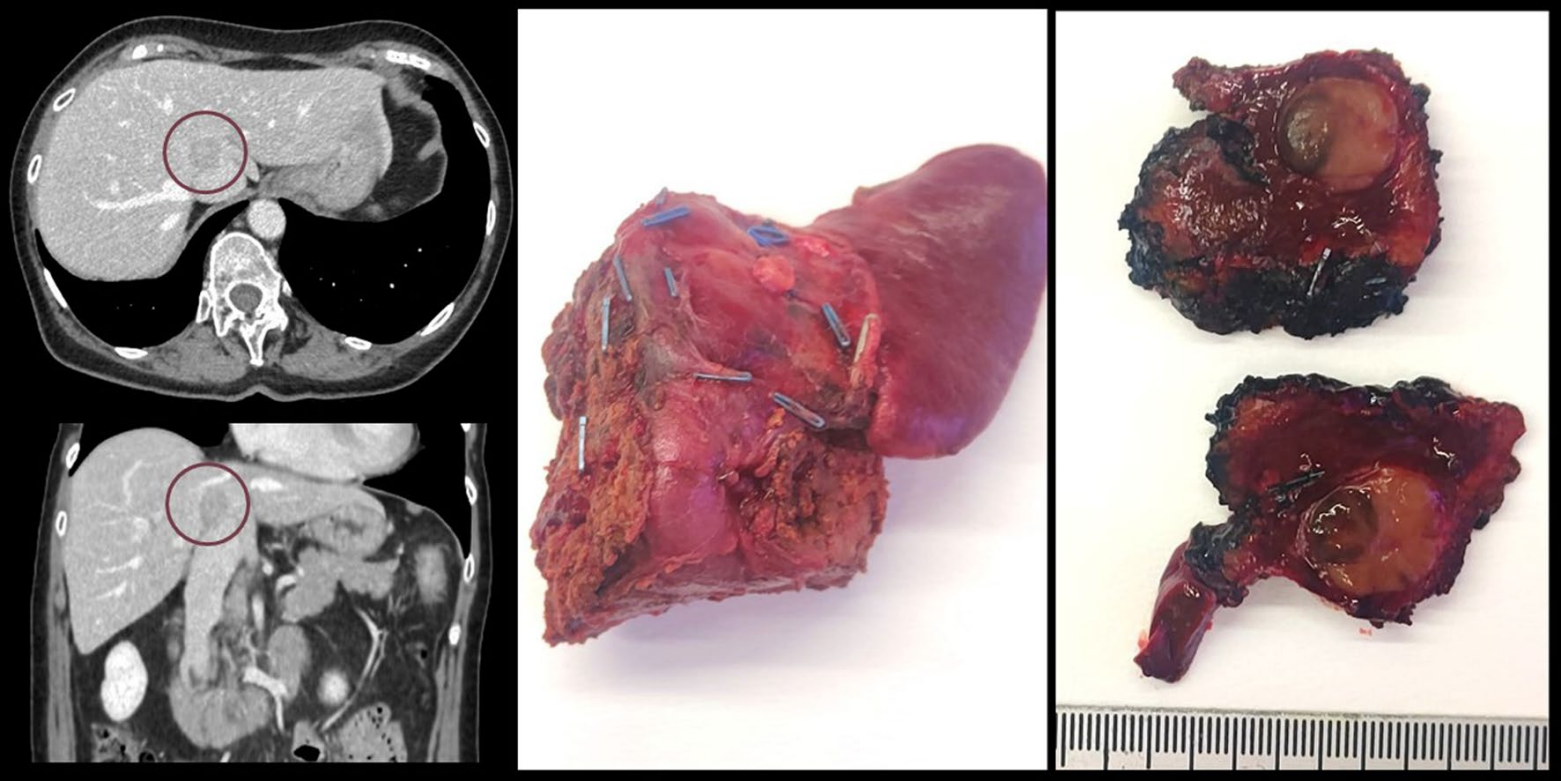
Preoperative CT scan and surgical specimen of case 15 from the surgery group; a liver tunnel for a single LMUM located in segment 1

Regarding the 15 intraoperatively resectable cases, 8 (53%) had to be converted to laparotomy, mostly at the beginning of our surgical experience. Four patients received a resection of a single lesion, seven underwent multiple hepatic resections, three underwent multiple hepatic resection with radiofrequency ablations, and one underwent a right hepatectomy. Figure 1 shows the surgical specimen of case 15, a liver tunnel for a single LMUM located in segment 1. On histology, all resections were confirmed as LMUM. All resections had clear margins. Minor postoperative complications occurred in 3 (20%) cases of hepatic resection, with no major complications and no complications in the staging alone group. Postoperative length of stay was predictably longer in the surgery group than in the staging alone group (median 6 days versus 3.5 days, respectively, *p* = 0.009). No readmissions or postoperative mortality occurred.

LMUM treatment of the staging alone group consisted of tebentafusp in two cases, immunotherapy in two cases, TACE + tebentafusp in one case, TACE + immunotherapy in one case, and fotemustine in one case.

### Long-Term Follow-up

**Fig. 2:**
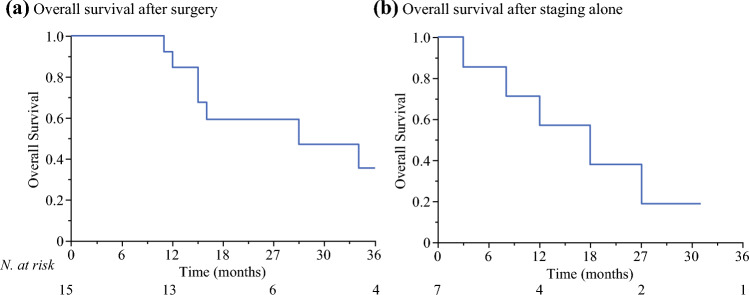
Kaplan–Meier curve of overall survival of patients who underwent curative-intent surgery (**a**) and staging alone (**b**)

After a median follow up of 86 (14–86) months, 14 patients died: 9/15 in the surgery group (7 LMUM-related deaths, 2 owing to other causes) and 5/7 in the staging alone group (all LMUM-related deaths). The median OS of the surgery group (Fig. 2a) was 27 (15–47) months, with a 1-year OS of 85% and a 2-year OS of 59%. The median OS of the staging alone group (Fig. 2b) was 15 (8–21) months, with a 1-year OS of 50% and a 2-year OS of 0.

The long-term outcomes of the 15 patients in the surgery group are presented in Table 2. Seven experienced recurrence at a median of 4 (1.5–21.5) months from surgical resection. Median DFS was 28 (4–38) months; four patients had recurrence detected at 4 months from resection, i.e., at the first oncological follow-up. Moreover, 1-year and 2-year DFS were 67% and 33%, respectively.

**Table 2:** Characteristics and follow-up of the resected patients

No.	Time from primary to LM, mo	No. LM	Max size LM, mm	Status	OS	Recurrence	TTR, mo	Recurrence site	Recurrence treatment
1	228	8	35	Dead	16	No			
2	36	2	12	Dead	15	Yes	4	Liver + lung	Fotemustine
3	72	2	9	Alive	86	Yes	28	Liver	Surgery
4	34	1	38	Dead	11	No			
5	204	2	50	Dead	51	Yes	38	Liver + bone	Anti-CTLA4 + TACE
6	42	2	9	Dead	15	Yes	4	Bone	Anti-CTLA4
7	61	1	12	Dead	47	Yes	4	Liver	Surgery
8	47	3	15	Dead	34	Yes	4	Liver + lung	Anti-PD1
9	8	6	10	Dead	27	Yes	4	Liver	Anti-CTLA4 + TACE
10	7	6	35	Dead	12	No			
11	35	1	14	Alive	21	No			
12	59	1	40	Alive	19	No			
13	15	1	15	Alive	14	No			
14	101	5	38	Alive	6	No			
15	38	1	27	Alive	6	No			

*LM* liver metastases, *mo* months, *TTR* time to recurrence, *TACE* transarterial chemoembolization

Recurrence involved the liver in six out of seven cases (three liver-limited, three liver and extrahepatic) and bone alone in one case. No local recurrences were observed. Two patients were treated with surgery, while the other five underwent systemic therapy, which included TACE in two cases. The median PRS was 26 (13–43) months.

## Discussion

In this monocentric series, patients who underwent curative-intent resection for liver metastases from uveal melanoma had longer overall survival than preoperatively resectable patients who were found to have unresectable disease at surgery and thus underwent other treatments for their LMUM.

The rarity and aggressive behavior of LMUM pose significant challenges in their management. Several treatment options exist; however, the oncological results of systemic and locoregional therapies are suboptimal.^4^ The median OS for locoregional therapies in liver-limited LMUM is reportedly around 18 months,^14^ reaching as far as 21.7 months with isolated hepatic perfusion (IHP) in the SCANDIUM trial.^15^ Systemic therapy is also ineffective, with a median OS of 15.1 months for tebentafusp in the IMCgp100-202 trial^5^ and 12.7 months for the combination of ipilimumab and nivolumab in the GEM-1402 study.^16^ In the FOCUS trial of percutaneous hepatic perfusion (PHP) versus best alternative care, the median OS was 20.5 months for PHP versus 14 months for other therapies.^17^

Surgical management of LMUM remains controversial owing to the risk of miliary disease discovery during exploration,^10^ which results in the procedure being aborted and the risk of futility owing to the development of early recurrence. Despite these considerations, data from literature consistently suggest that curative-intent surgery and/or ablation can provide a survival advantage in these patients. The landmark paper by Mariani et al. in 2009,^8^ describing 255 liver resections for LMUM, showed a median OS of 27 months for R0 resections. In their series, predictors of OS were an interval between primary tumor and LMUM > 24 months, R0 resection, maximum of four liver lesions, and no miliary disease. Similar results were evidenced by subsequent series, as reported by recent literature review by Trivedi et al.^18^

Our results are in line with those previously obtained by other centers. While not statistically significant, likely owing to the small sample size, the surgery group and staging only group had relevant differences; patients who were able to undergo surgical resection had a longer lead time between primary and LMUM (median 44.5 months versus 28 months in the staging alone group). In addition, their lesions were smaller and lower in number and had less bilobar involvement (21% versus 57 % in the staging alone group).

OS was significantly longer after surgery than after staging alone, with a median OS of 27 months versus 15 months. This is mostly related to selection bias; by definition, patients who could not be resected had more advanced disease than patients who could be resected. However, the reason most patients could not be resected was owing to miliary disease in the form of small ubiquitous lesions, while 33% of patients in the surgery group also showed intraoperative non-concordance with preoperative imaging but could be resected owing to bulkier, confined lesions. It could thus be speculated that the survival advantage may not only be owing to better biology alone but to the higher efficacy of liver resection compared with other treatments. A similar comparison was reported by Trivedi et al. in which survival after liver resection was also significantly longer than after laparoscopy and liver biopsy alone owing to intraoperative unresectability.^19^

Concordance between preoperative staging and intraoperative findings was 45% in our series, with 23% of patients having more lesions than previously detected and 27% of patients with miliary disease. These findings are similar to those reported by Servois et al.,^10^ who reported diffuse miliary disease in 3/12 (25%) patients who underwent surgical exploration with the intent of achieving curative resection, which could not be performed. To avoid futility, we should improve the accuracy of preoperative staging. All patients in our series underwent MRI less than 1 month before surgery; however, this was not sufficient. A recent meta-analysis of 27 studies has highlighted the accuracy of FDG-PET for the staging of metastasis UM.^20^ In their results, the value of FDG uptake appears to be prognostic for patient survival. Implementation of FDG-PET may help avoiding futile surgical explorations; however, it must be noted that most of the futility is owing to miliary disease, i.e., diffuse lesions < 2–3 mm in diameter, which may not be detected even with FDG-PET owing to limited spatial resolution.

It must be noted that none of the patients who only underwent staging had a postoperative complication and all were able to receive other treatments. It is well-known in literature that staging laparoscopy is safe and is routinely employed in tumors at high risk of peritoneal carcinomatosis before the start of systemic therapy, e.g., gastric and pancreatic cancer.^21,22^ For these reasons, exploratory laparoscopy may be employed in LMUM in all cases that may have a surgical chance without compromising patient safety or future treatment options.

The incidence of postoperative complications in our series was low, even among patients who underwent surgical resection, with no major complications (Clavien–Dindo grade ≥ 3) and no postoperative mortality. The safety of hepatic surgery is supported by large series, with reported overall morbidity between 15 and 30% and mortality between 0.5 and 2%.^23–25^ Thus, in addition to providing apparently superior oncological results, surgery may also have a non-inferior safety profile than other therapies for LMUM. Indeed, both PHP and IHP are highly invasive procedures that require venovenous bypass and complex anesthesiological management. For example, the FOCUS trial reported serious adverse events in 51.2% of patients receiving PHP.^17^ In the SCANDIUM trial, one treatment-related death occurred in the IHP group owing to hepatic artery dissection.^15^ One treatment-related death was also reported by Carle et al. after TACE,^26^ in addition to 17 (12.3%) postembolization syndromes after 138 TACEs.

The recurrence rate after curative-intent surgery was high, with 33% DFS at 2 years. All recurrences involved the liver, except one, which was limited to the bone. Four patients underwent surgery or locoregional therapies in combination with immunotherapy, while three patients underwent systemic therapy alone. Median postrecurrence survival was high, highlighting limited aggressiveness.

This study has several limitations. It is a monocentric case series with a limited number of cases spanning several years; therefore, a larger number of patients is needed to confirm the favorable outcomes of patients receiving radical surgery. However, our results are in line with most published literature and confirm a potential benefit of surgery with respect to palliative treatments in this setting.

In conclusion, surgical exploration for selected patients with LMUM was associated with a high resection rate and low rate of postoperative complications, both in the case of exploration alone and in the case of hepatic resection. Survival after hepatic resection was in line with previous experiences and higher than the reported results for non-surgical therapies for LMUM. Our study suggests that laparoscopic exploration and aggressive surgical management of LMUM may be warranted in selected cases.

## Data Availability

The data, code, and other materials are available from the corresponding author upon reasonable request.
